# Determinants of phosphatidylinositol-4-phosphate 5-kinase type Iγ90 uropod location in T-lymphocytes and its role in uropod formation

**DOI:** 10.7717/peerj.131

**Published:** 2013-08-29

**Authors:** Lucia Mathis, Sarah Wernimont, Sarah Affentranger, Anna Huttenlocher, Verena Niggli

**Affiliations:** 1 Institute of Pathology, University of Bern, Bern, Switzerland; 2 Departments of Medical Microbiology and Immunology and Pediatrics, University of Wisconsin, Madison, WI, United States of America

**Keywords:** T cell, Phosphatidylinositol-4-phosphate 5-kinase type I (PIPKI)γ90, PIPKIγ87, Uropod, Flotillin

## Abstract

We have previously identified phosphatidylinositol-4-phosphate 5-kinase type I (PIPKI)γ90 as a T cell uropod component. However, the molecular determinants and functional consequences of its localization remain unknown. In this report, we seek to better understand the mechanisms involved in PIPKIγ90 uropod targeting and the role that PIPKIγ90 plays in T cell uropod formation. During T cell activation, PIPKIγ90 cocaps with the membrane microdomain-associated proteins flotillin-1 and -2 and accumulates in the uropod. We report that the C-terminal 26 amino acid extension of PIPKIγ90 is required for its localization to the uropod. We further use T cells from PIPKIγ90^−/−^ mice and human T cells expressing a kinase-dead PIPKIγ90 mutant to examine the role of PIPKIγ90 in a T cell uropod formation. We find that PIPKIγ90 deficient T cells have elongated uropods on ICAM-1. Moreover, in human T cells overexpression of PIPKIγ87, a naturally occurring isoform lacking the last 26 amino acids, suppresses uropod formation and impairs capping of uropod proteins such as flotillins. Transfection of human T cells with a dominant-negative mutant of flotillin-2 in turn attenuates capping of PIPKIγ90. Our data contribute to the understanding of the molecular mechanisms that regulate T cell uropod formation.

## Introduction

Migrating leukocytes establish a polarized morphology with an actin-rich leading edge and a uropod protruding from the trailing edge. This polarization is essential for efficient crawling. The uropod is a plasma membrane protrusion that contains specific organelles along with cytoskeletal, adhesion and signaling proteins (Serrador et al., 1997; Sánchez-Madrid & Serrador, 2009). Flotillins, membrane microdomain scaffolding proteins, are also enriched in leukocyte uropods and are involved in uropod formation (Rossy et al., 2009; Ludwig et al., 2010; Affentranger et al., 2011; Baumann, Affentranger & Niggli, 2012). Recent in vivo data using inhibition of uropod formation by suppressing Rho-kinase activity suggest that the uropod is especially important for T cell migration through constricted spaces (Soriano et al., 2011).

During leukocyte migration, Rac2 mediated actin polymerization at the leading edge drives cell protrusion, while RhoA activation controls myosin II dependent uropod retraction (Xu et al., 2003). This process of protrusion and retraction is exquisitely coordinated to support efficient cell migration. Phosphoinositides are important signaling lipids regulating these processes. Research has focused especially on phosphatidylinositol (4,5) bisphosphate (PI(4,5) P_2_) and phosphatidylinositol (3,4,5) trisphosphate. Production of the former lipid is regulated by phosphatidylinositol-4-phosphate 5-kinase type I (PIPKI) isoforms and that of the latter lipid by phosphoinositide 3-kinase (Ward, Westwick & Harris, 2011; Sun et al., 2013). Previously, we identified the enzyme PIPKIγ90 enriched in the neutrophil and the T cell uropod (Lokuta et al., 2007). PIPKIγ90 has been implicated in the regulation of integrin activity and endocytosis (Calderwood et al., 2004; Bairstow et al., 2006; Sekine et al., 2007). Two isoforms of PIPKIγ that differ by a 26 amino acid C-terminal extension are expressed in T cells; a 635 amino acid (87 kDa) isoform, PIPKIγ87, and a 661 amino acid (90 kDa) isoform, PIPKIγ90 (Wernimont et al., 2010). Neutrophils lacking both PIPKIγ isoforms have impaired neutrophil adhesion and trafficking to sites of infection. PIPKIγ87 lacks the sequence required for uropod targeting as observed in neutrophils (Xu et al., 2010). In T cells, siRNA knockdown of both PIPKIγ isoforms is associated with decreased integrin mediated T cell adhesion in response to chemokine signaling (Bolomini-Vittori et al., 2009), whereas T cells from PIPKIγ90 deficient mice have increased integrin mediated adhesion in response to T cell receptor-mediated signaling (Wernimont et al., 2010), indicating that the two isoforms have distinct functions.

In this report, we seek to better understand how uropod location of PIPKIγ90 is controlled in T cells, and its role in T cell uropod formation. By over-expressing GFP-tagged PIPKIγ in murine D10 T cells and in human freshly isolated T cells, we find that the 26 amino acid extension of PIPKIγ90 is required for its localization to the uropod. We moreover observe that PIPKIγ90 deficient murine T cells and human T cells expressing kinase-dead PIPKIγ90 have elongated uropods. These findings show that similar mechanisms control uropod targeting of PIPKIγ90 in both neutrophils (Xu et al., 2010) and T cells (this work), and that PIPKIγ90 is involved in uropod retraction in both cell types. We also provide novel data on the interrelationship between flotillins and PIPKIγ90.

## Materials and Methods

### Ethics Statement

We received approval for this study from the University of Wisconsin School of Medicine and Public Health Institutional Animal Care and Use Committee (protocol number MO1570-0-06-07).

### Mice

PIPKIγ90^−/−^ mice were generated as previously described and fully backcrossed onto the C57/Black6 background (Wernimont et al., 2010).

### Reagents

Antibodies for immunofluorescence and immunoblotting:

Phospho ezrin (Thr567)/radixin (Thr564)/moesin (Thr558) (P-ERM) (Cell Signaling Technology, Beverly, MA); flotillin-2 (Sigma, F-1680).

Recombinant proteins: human stromal cell-derived factor-1 (SDF-1 alpha), recombinant murine intercellular adhesion molecule-1 (rmICAM-1) and recombinant human ICAM-1 (rhICAM-1) were purchased from R and D systems (Minneapolis, MN).

Gey’s solution contained 138 mM NaCl, 6 mM KCl, 100 µM EGTA, 1 mM Na_2_HPO_4_, 5 mM NaHCO_3_, 5.5 mM glucose and 20 mM HEPES (pH 7.4).

### Isolation, cell culture and retroviral transfection of murine T cells

Murine D10 T cells (ATCC) were retrovirally transduced with GFP or GFP-PIPKIγ87, GFP-PIPKIγ90 or GFP-PIPKIγ90 kinase dead (KD) as previously described (Lokuta et al., 2007; Wernimont et al., 2010). Following retroviral transduction, fluorescent cells were sorted by FACS. T cells were maintained in complete RPMI supplemented with IL-2 (Chiron).

Single cell suspensions of primary mouse T cells were made from lymph nodes and spleen from control and PIPKIγ90 knockout mice that were between 6 and 10 weeks of age. Following red blood cell lysis, CD4^+^ T cells were isolated from cell suspension by negative selection and automacs sorting (Miltenyi). Isolated CD4^+^ T cells were then stimulated 1:1 with anti-CD3/CD28 coated beads according to the manufacturer’s instructions (Invitrogen) and maintained in RPMI supplemented with IL-2 (Chiron). These anti-CD3/CD28 bead activated cells were used for in vitro assays days 7 to 10 following isolation.

### Isolation and transient transfection of human T cells

T-lymphocytes were isolated from buffy coats of healthy donor blood using the Pan T Cell Isolation Kit II (Miltenyi Biotec) and separation on LD columns (Miltenyi Biotec) according to the manufacturer’s instructions. The buffy coats were obtained from the Central Laboratory of the Swiss Red Cross, Bern, Switzerland. For details see Affentranger et al. (2011).

For transient transfections, 5 × 10^6^ freshly isolated T cells were resuspended in 100 µl human T cell nucleofector solution (Amaxa, Köln, Germany) diluted 1:2 with PBS and 1–2 µg of DNA per construct was added, followed by nucleofection (Amaxa Nucleofector, program U-14). Constructs encoding for flotillin-2 and -1 C-terminally tagged with mCherry were prepared as described (Rossy et al., 2009; Affentranger et al., 2011). Constructs encoding for PIPKIγ90 and PIP5Kγ87 N-terminally tagged with GFP and subcloned in a pcDNA3.1 vector were prepared as described (Lokuta et al., 2007). The point mutation D253A was introduced into the wild type enzyme using the Quick Change Mutagenesis kit from Stratagene.

Immediately after transfection, 500 µl of RPMI with 20% FCS was added and the cells were transferred to a prewarmed 12-well plate containing 2.5 ml of RPMI with 20% FCS, followed by incubation at 37°C in a CO_2_ incubator for 4–6 h. Transfected cells were subsequently washed and resuspended in Gey’s solution.

### Immunofluorescence staining of murine T cells

Murine wild type and PIPKIγ90^−/−^ T cells were allowed to migrate on rmICAM-1 coated coverslips for 15 min at 37°C prior to fixing with 3% paraformaldehyde (PFA) (Electron Microscopy Services) for 15 min at 25°C. Cells were permeabilized with 0.2% Triton X-100, blocked in goat serum and stained with anti-P-ERM antibody (Santa Cruz), DAPI and rhodamine phalloidin (Invitrogen) along with FITC conjugated anti-rabbit secondary antibodies (Jackson Labs). Images were acquired on a laser scanning confocal microscope (Olympus) using a 60 × Plan Apo/1.45 oil immersion objective with a 10× zoom factor and captured into Fluoview software (FV10-ASW version 01.07; Olympus).

### Immunofluorescence staining of human T cells

The transfected cells were resuspended in Gey’s medium containing 1 mM MgSO_4_, 1.1 mM CaCl_2_ and plated on glass coverslips coated with 3 µg hrICAM-1/ml, incubated at 37°C and 5% CO_2_ for 45 min followed by addition of SDF-1 (40 ng/ml) and a further incubation for 15 min. Cells were fixed with 3.7% PFA for 15 min followed by staining with rhodamine phalloidin (Molecular Probes) and Hoechst 33342 dye (Sigma) or with an antibody recognizing flotillin-2 as described (Affentranger et al., 2011). Pictures were acquired on a confocal microscope (Olympus FV 1000) equipped with an 60 × Plan Apo/1.45 oil immersion objective and captured into Fluoview software (FV10-ASW version 01.7).

Flotillin- and PIPKIγ90-enriched aggregates located at the plasma membrane of at least 1 µm or larger (maximally approximately 4 µm) were defined as “caps”. For analysis of shape and protein localization, 50–100 T cells were analysed per sample and experiment.

### Statistical analysis

Data were analyzed with the Graph Pad Prism software (version 5.04) using ANOVA with Tukey’s post hoc testing or with the student’s t-test, depending on the experiment, as detailed in the figure legends. *P* values <0.05 were considered significant. Data correspond to the mean ± sem.

## Results

### PIPKIγ90 specifically localizes to the T cell uropod independent of its kinase activity but dependent on its 26 residue C-terminal extension

**Figure 1:**
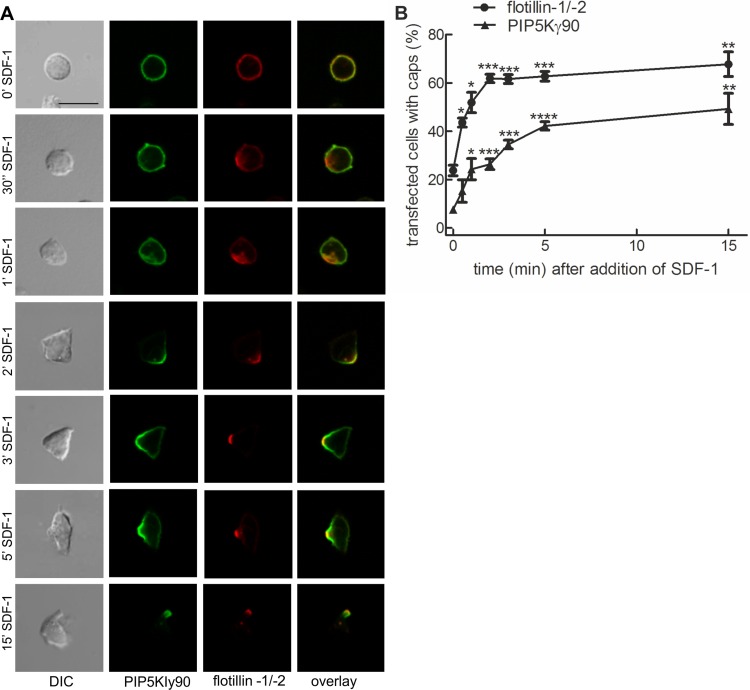
PIPKIγ90 cocaps with flotillins in human T cells during SDF-1-induced uropod formation. T cells were co-transfected with GFP-tagged wild type PIPKIγ90, flotillin-1-mCherry and flotillin-2-mCherry, followed by incubation at 37°C for 30 min in suspension. Note that singly expressed flotillin-1 or -2 do not cap (Affentranger et al., 2011). Cells were then treated with SDF-1 (40 ng/ml) for the indicated times and fixed in suspension with 3.7% PFA. (A) Fluorescence pictures. Scale bar 10 µm. (B) Quantitative evaluation of (A). The percentage of transfected cells with capped flotillins or PIPKIγ90 were derived from *n* = 3 independent experiments (mean ± sem) (^∗^*p* < 0.05, ^∗∗^*p* < 0.01,^∗∗∗^*p* < 0.001, ^∗∗∗∗^*p* < 0.0001 for differences to cells not exposed to SDF-1, obtained with Anova and Tukey’s multiple comparison test). 50 cells were counted per sample and experiment.

Previous work from our lab with murine T cells has identified PIPKIγ90 as a uropod component in neutrophils and murine T cells (Lokuta et al., 2007). We studied kinetics of uropod recruitment of PIPKIγ90 in human freshly isolated T cells stimulated with the chemokine SDF-1. As known T cell uropod components we used the the raft proteins flotillin-1 and -2 (Affentranger et al., 2011; Baumann, Affentranger & Niggli, 2012). As shown in Fig. 1A, flotillins and PIPKIγ90 are mostly uniformly distributed in the spherical resting cells, but cap rapidly and arrive together in the uropod upon chemokine addition. Capping especially of flotillins precedes formation of the uropod. Capping of PIPKIγ90 lags slightly behind that of flotillins (t1/2 for flotillin: approximately 1 min; t1/2 for PIPKIγ90: approximately 2 min), and the percentage of fully polarized cells with flotillin caps is higher than that of cells with PIPKIγ90 caps (Fig. 1B).

**Figure 2:**
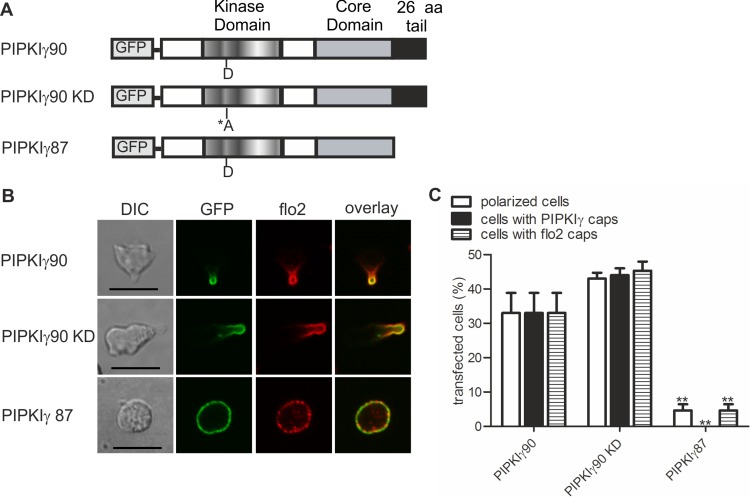
Determinants of uropod targeting of PIPKIγ90 in human T cells. (A) Scheme showing the structure of PIPKIγ90 and of the constructs used in this work. (B, C) Human freshly isolated T cells were transiently transfected with GFP-tagged wild type or the indicated mutant PIPKIγ constructs, followed by plating on ICAM-1, and, after a 45 min incubation at 37°C, stimulation with 40 ng SDF-1/ml for 15 min. Cells were then fixed with PFA and stained for endogenous flotillin-2 (flo2) with a rabbit polyclonal antibody. (B) Representative immunofluorescence pictures showing the location of GFP tagged wild type and mutant PIPKIγ (green) and flotillin-2 (red). Scale bar 10 µm. (C) Quantitative evaluation of the experiment shown in (B) concerning the % of transfected polarized cells and the % of transfected cells with PIPKIγ and flotillin-2 (flo2) caps. Mean ± sem of 3 experiments (^∗∗^*p* < 0.01 as compared to cells transfected with wild type PIPKIγ90 obtained by ANOVA and Tukey’s multiple comparison test). 50 cells were counted per sample and experiment.

We now studied mechanisms involved in uropod targeting of this enzyme by expressing GFP-tagged wild type and mutated PIPKIγ90 in freshly isolated human T cells. The constructs used in this study are shown in Fig. 2A. We found that wild type PIPKIγ90 locates to the uropod of freshly isolated human T cells randomly migrating on ICAM-1 in the presence of SDF-1 (Fig. 2B), as well as in murine D10 T cells migrating on ICAM-1 (Fig. S1). In neutrophils the PIPKIγ90 kDa isoform specifically localizes to the uropod while the 87 kDa isoform is uniformly distributed around the cell cortex (Lokuta et al., 2007; Xu et al., 2010), suggesting that its localization is regulated by the 26 residue C-terminal extension.

We also obtained comparable results in human T cells (Figs. 2B and 2C). Kinase-dead PIPKIγ90 also localizes to the T cell uropod indicating that localization is not kinase dependent (Figs. 2B and 2C). Similar data were obtained for murine D10 T cells (Fig. S1). In human T cells expressing GFP-tagged PIPKIγ87 and plated on ICAM-1 in the presence of the chemokine SDF-1, formation of contracted uropods was almost completely abolished. Capping of the uropod component flotillin-2 was also markedly reduced (Figs. 2B and 2C). Similarly uropod capping of the adhesion receptor PSGL-1 was inhibited (V Niggli and S Affentranger, unpublished data). This inhibitory effect was less striking in the murine D10 T cell clone (Fig. S1), possibly because these cells are already activated and polarized in the absence of stimuli, whereas freshly isolated T cells are mainly spherical in the absence of chemokine (Fig. 1A). In the latter situation, transfection with the PIPKIγ87 construct prevents polarization whereas for the D10 T cell clone already existing uropods may be resistant to disruption. Our findings suggest a role of PIPKIγ90 in uropod formation, possibly as a scaffolding protein.

### Transfection of human T cells with a dominant-negative mutant of flotillin-2 impairs capping of PIPKIγ90

**Figure 3:**
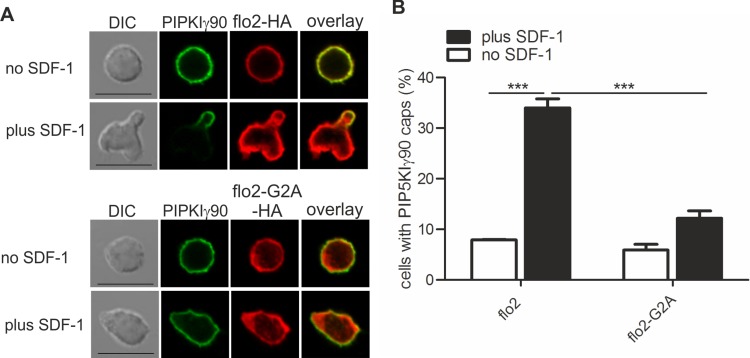
Coexpression of dominant-negative flotillin-2-G2A inhibits capping of GFP-PIPKIγ90 in human T cells. T cells were co-transfected with wild type flotillin-2-HA and GFP-tagged wild type PIPKIγ90 or flotillin-2-G2A-HA and GFP-tagged wild type PIPKIγ90 and incubated at 37°C for 30 min. Cells were then stimulated with SDF-1 (40 ng/ml) for 15 min, fixed with 3.7% PFA (final concentration) in suspension and stained for HA. (A) Fluorescence pictures. Scale bar 10 µm. (B) Quantitative evaluation of A: % of cells with PIPKIγ90 caps in transfected cells were derived from *n* = 3 independent experiments (mean ± sem) (^∗∗∗^*p* < 0.001 obtained by ANOVA and Tukey’s multiple comparison test). 100 cells counted per sample.

We have previously shown that flotillins are involved in T cell uropod formation and that transfection of human T cells with a dominant-negative mutant of flotillin-2 (flotillin-2-G2A) impairs uropod formation and capping of uropod proteins such as PSGL-1 (Affentranger et al., 2011). Similarly, transfection of human T cells with this mutant significantly reduced chemokine-induced capping of PIPKIγ90 by 64 ± 2% (*n* = 3, *p* < 0.001) (Figs. 3A and 3B), suggesting that flotillins and PIPKIγ90 cooperate as scaffolding proteins in the uropod. Cell polarity was also inhibited by 41 ± 2% (*n* = 3, *p* < 0.01) in SDF-1-stimulated cells, in agreement with previous data on human T-lymphoblasts (Affentranger et al., 2011).

### PIPKIγ90^−/−^ murine T cells or human T cells expressing kinase-dead PIPKIγ90 have elongated uropods

**Figure 4:**
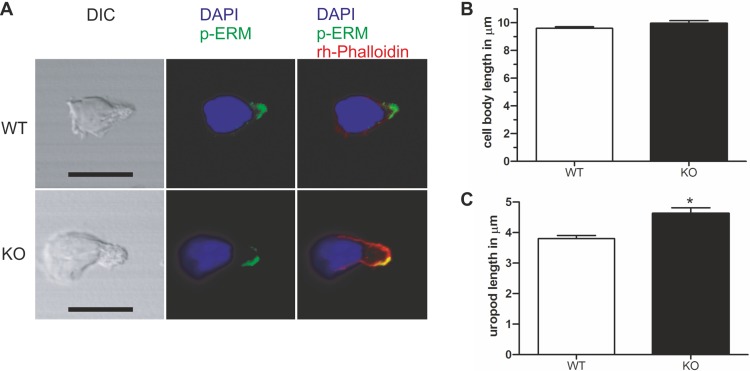
Murine PIPKIγ90^−/−^ T-cells have elongated uropods. Wild type and knockout murine T cells were fixed while migrating on an ICAM-1 coated coverslip and stained with DAPI, rhodamine phalloidin, and antibodies specific for P-ERM. (A) Representative immunofluorescence pictures showing the location of DAPI (blue), F-actin (red) and P-ERM (green). Scale bar 10 µm. (B) Quantification of cell body length defined as the distance in µm from the leading edge to the trailing edge of the nucleus. (C) Quantification of uropod length-defined as the length in µm from the trailing edge of the nucleus to end of the uropod which is enriched in P-ERM. ^∗^ = *p* < 0.001 by students t test (mean ± sem from 40 cells per condition from 3 independent experiments).

Given the specific localization of PIPKIγ90 to the T cell uropod, and the negative impact of PIPKIγ87 on uropod formation in human T cells, we explored the role of PIPKIγ90 in uropod formation using CD4^+^ T cells from PIPKIγ90^−/−^ mice. These cells were fixed while migrating on ICAM-1 and stained with antibodies recognizing phosphorylated ERM proteins as uropod markers along with DAPI and rhodamine phalloidin to visualize the nucleus and actin cytoskeleton respectively. As shown in Fig. 4, T cells from PIPKIγ90 knockout mice were longer than control cells. Quantification of the cell body length, defined as the length from the leading edge to the trailing edge of the nucleus, was not different between control and knockout cells. Rather, the difference in cell length was attributable to an approximately 30% increase in uropod length (measured from the trailing edge of the nucleus to the end of the uropod).

**Figure 5:**
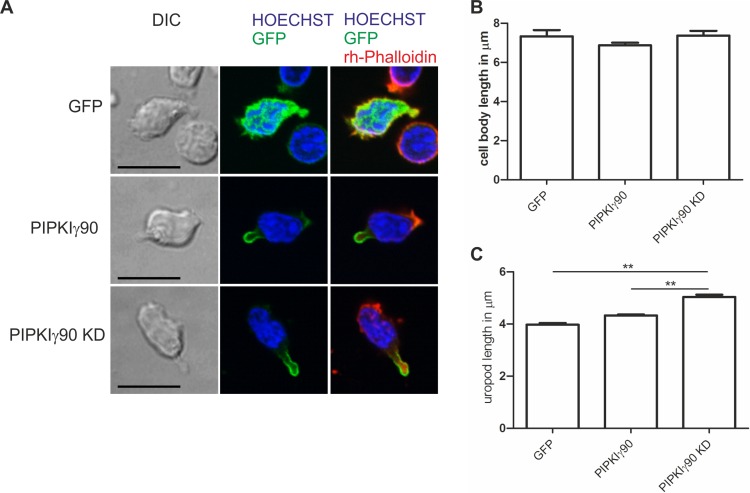
Transfection with kinase-dead PIPKIγ90 induces uropod elongation in human T cells. Human T cells were transiently transfected with GFP, GFP tagged wild type PIPKIγ90, or GFP tagged kinase dead PIPKIγ90 (PIPKIγ90 KD). Cells were incubated at 37°C for 30 min, incubated on ICAM-1-coated cover slips at 37°C for 45 min, stimulated with SDF-1 (40 ng/ml) for 15 min, fixed with 3.7% PFA and stained with Hoechst dye and rhodamine phalloidin. (A) Representative immunofluorescence pictures showing the location of the PIPKIγ constructs (green), Hoechst dye (blue) and F-actin (red). Scale bar 10 µm. (B) Quantification of cell body length defined as the distance in µm from the leading edge to the trailing edge of the nucleus. (C) Quantification of uropod length defined as the length in µm from the trailing edge of the nucleus to the end of the uropod. Data shown in (B) and (C) were derived from a total of 60 cells per condition obtained in 3 independent experiments (mean ± sem) (^∗∗^*p* < 0.01 obtained by ANOVA and Tukey’s multiple comparison test).

As shown in Fig. 2, expression of kinase-dead PIPKIγ90 in human T cells did not affect cell polarization and capping of uropod proteins such as flotillins. However during SDF-1-stimulated migration on ICAM-1, freshly isolated human T cells transfected with kinase-dead PIPKIγ90 had elongated uropods as compared to cells expressing wild type PIPKIγ90 or EGFP. We observed a 27% increase in uropod length when comparing human T cells transfected with EGFP to cells expressing kinase-dead PIPKIγ90 (Figs. 5A–5C).

## Discussion

The work presented here shows that PIPKIγ90 is specifically localized to the T cell uropod, whereas the PIPKIγ87 isoform shows a diffuse location, implicating the 26 amino acid C-terminal extension in uropod targeting, similar to findings in neutrophils. Additionally, we show that PIPKIγ90 regulates adhesion of the T cell uropod since in its absence murine T cells have elongated uropods, very likely due to impaired de-adhesion of the rear of the cell. Comparable findings were obtained for human T cells transfected with kinase dead PIPKIγ90. Moreover our data suggest novel scaffolding functions of PIPKIγ90 in the uropod.

Our data indicate divergent functions for the 87 and 90 kDA PIPKIγ isoforms. This is not entirely unexpected, since previous work has shown that they differently regulate calcium signaling and integrin mediated adhesion. For instance, PIPKIγ87 is the isoform responsible for generating the PI(4,5)P_2_ required for calcium signaling (Wang et al., 2004), whereas specific knockdown of PIPKIγ90 actually increases adhesion of T cells to ICAM-1 (Wernimont et al., 2010).

We investigated the functional role of PIPKIγ90 in uropod formation and show that murine T cells lacking this enzyme or human T cells expressing kinase-dead PIPKIγ90 have elongated uropods when migrating on ICAM-1, comparable to differentiated neutrophil-like HL-60 cells transfected with kinase-dead PIPKIγ90 (Lokuta et al., 2007). This suggests that de-adhesion of uropods is impaired in cells lacking PIPKIγ90 or expressing the kinase-dead mutant which may interfere with the function of the endogenous enzyme, for example by displacing it from interaction partners. Localized increases in PI(4,5)P_2_ in the rear of the cell mediated by PIPKIγ could lead to localized activation of ERM proteins, resulting in F-actin-membrane linkage and enhanced Rho activity inducing uropod contraction (Ivetic & Ridley, 2004). Knockdown of PIPKIγ could thus result in a reduction of uropod contractility and therefore a decrease in uropod detachment.

Interestingly, expression of PIPKIγ87, which lacks the uropod targeting domain, in human T cells resulted in almost complete impairment of uropod formation and capping of uropod proteins (Fig. 2). These findings are similar to those previously obtained for the isoform PIPKIβ in neutrophil-like HL-60 cells (Lacalle et al., 2007). There, expression of kinase-dead PIPKIβ induced cell elongation, whereas expression of a truncated form of PIPKIβ lacking the last 83 C-terminal amino acids, which does not locate to the uropod, had a much stronger phenotype and reduced cell polarization by about 80%. Lacalle et al. (2007) argue that the truncated PIPKIβ may act by sequestering proteins required for uropod formation and prevent their interaction with endogenous PIPKIβ, thus strongly impairing uropod formation. This could also explain the strong effects of the mutant PIPKIγ87 observed in this work. In the murine T cells lacking PIPKIγ90, other isoforms such as PIPKIβ could compensate for this loss and explain the relatively mild phenotype. Transfection with the kinase-dead PIPKIγ90 may only incompletely interfere with activity of the endogenous PIPKIγ isoform. Both PIPKIγ90 and PIPKIβ may thus act as scaffolds to organize signaling at the rear of polarized leukocytes, together with the raft-associated flotillins. We show that flotillins, directly or indirectly, also are involved in PIPKIγ90 uropod targeting (Fig. 3). PIPKIγ90 and PIPKIβ have divergent C-terminal amino acid sequences and may be targeted to the uropod via different mechanisms. For example, PIPKIγ90, but not PIPKIβ, interacts with talin as shown in vitro (Manes et al., 2010).

## Conclusions

This work identifies a region of PIPKIγ90 necessary for its uropod localization in T cells, shows that T cells lacking PIPKIγ90 or expressing kinase-dead PIPKIγ90 have increased uropod length similar to findings in neutrophils, implicates flotillins in uropod targeting of PIPKIγ90 and provides novel data on possible scaffolding functions of PIPKIγ.

**Abbreviations**
PI(4,5)P_2_: phosphatidylinositol 4,5 bisphosphate
PIPKI: phosphatidylinositol-4-phosphate 5-kinase type I
ICAM-1: intercellular adhesion molecule 1
KD: kinase-dead
P-ERM: phosphorylated ezrin/radixin/moesin proteins
PFA: paraformaldehyde
rhICAM-1: recombinant human ICAM-1
rmICAM-1: recombinant murine ICAM-1
SDF-1: stromal cell-derived factor-1

## Supplemental Information

10.7717/peerj.131/supp-1 Fig. S1 Determinants of uropod targeting of PIPKIγ90 in murine T cells Representative immunofluorescence pictures showing the localization of GFP-tagged wild type and mutant PIPKIγ (green) and phospho-ERM (red) in murine D10 T cells randomly migrating on ICAM-1. Scale bar represents 5 µm. Images are representative of 30 cells from 3 independent experiments.
