# Supplementary figures and images for: Determinants of phosphatidylinositol-4-phosphate 5-kinase type Iγ90 uropod location in T-lymphocytes and its role in uropod formation
Source: PeerJ. 2013 Aug 29;1:e131. doi: 10.7717/peerj.131 (PMC3757496; doi:10.7717/peerj.131)

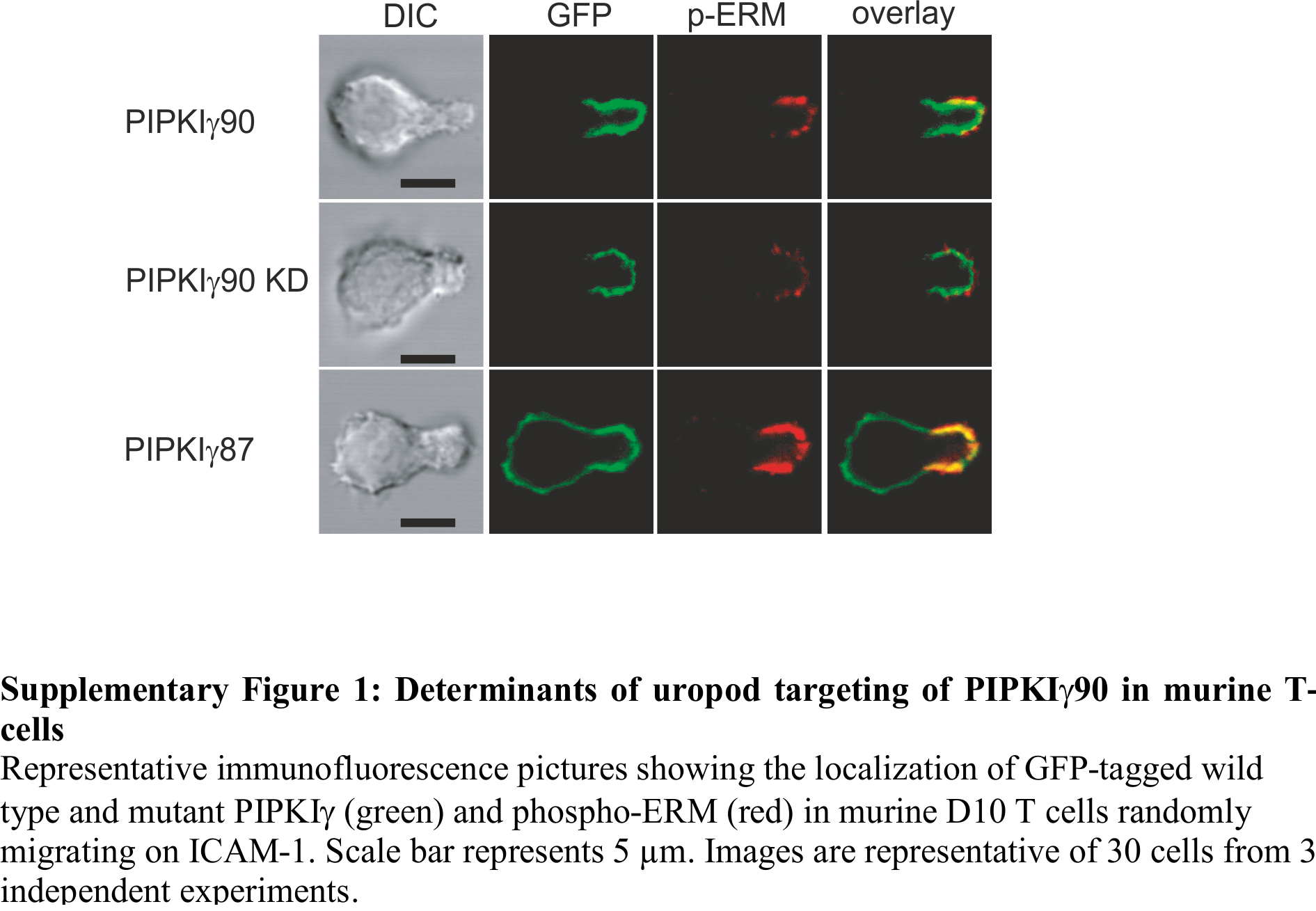

Supplement: Fig. S1 — Representative immunofluorescence pictures showing the localization of GFP-tagged wild type and mutant PIPKIγ (green) and phospho-ERM (red) in murine D10 T cells randomly migrating on ICAM-1. Scale bar represents 5 µm. Images are representative of 30 cells from 3 independent experiments. [file peerj-01-131-s001.png]
